# Supplementary material for: Systematic Identification of CpxRA-Regulated Genes and Their Roles in Escherichia coli Stress Response
Source: mSystems. 2022 Sep 7;7(5):e00419-22. doi: 10.1128/msystems.00419-22 (PMC9600279; doi:10.1128/msystems.00419-22)
Supplement: TABLE S1 [file msystems.00419-22-s0005.docx]

**Table S1　The data source of known CpxR binding sites**

| CpxR regulon | CpxR binding site |
| --- | --- |
| *acrD* | GTATAAAAGGGCATG([1](#_ENREF_1)) |
| *aer* | GTAACAGAGTGAAAG([2](#_ENREF_2)) |
| *alx (ygjT)* | GTAAAACAGTGTAAA([2](#_ENREF_2)) |
| *aroG* | GTAAAACCCCGTTTA([3](#_ENREF_3)) |
| *bacA* | GTAAAGCGGCGAAAA([2](#_ENREF_2)) |
| *bamE(smpA)* | GCAAAACGCCGTAAG([2](#_ENREF_2)) |
| *cpxR/cpxP1* | GTAAAACAACGTAAA([2](#_ENREF_2)) |
| *cpxR/cpxP2* | GCAAACATGCGTCAG([2](#_ENREF_2)) |
| *csgB1* | GTAACGCAGCGTTAA([2](#_ENREF_2)) |
| *csgB2* | GTAAAAAATTGTCCA([2](#_ENREF_2)) |
| *csgD1* | GTAACTAAATGTAAC([2](#_ENREF_2)) |
| *degP1 (htrA)* | GTAAAGACGAACAAT([2](#_ENREF_2)) |
| *degP2* | GTAAATTACCGTCAG([2](#_ENREF_2)) |
| *dgcZ (ydeH)* | GTCAATATTTTCATA([3](#_ENREF_3)) |
| *dsbC* | GTAAAGGCAACAAAG([4](#_ENREF_4)) |
| *efeU (ycdN)* | GGAATGTTTATAAAA([3](#_ENREF_3)) |
| *fabA* | GTAGAAGAAGGCAAA([5](#_ENREF_5)) |
| *fabB* | GTAAGGCTGCGCAAA([5](#_ENREF_5)) |
| *ftnB* | GTAAAAATATATAAA([3](#_ENREF_3)) |
| *ldtD* | GCAACTCCCTGAAAC([6](#_ENREF_6)) |
| *marR* | GCAAGTATAAGTCAA([7](#_ENREF_7)) |
| *motA* | GTAAAAAGACGTAAA([2](#_ENREF_2)) |
| *mviM* | GTAAATCACCGTCAA([2](#_ENREF_2)) |
| *mzrA (yqjA)* | GTCAGCCTGTGTAAA([3](#_ENREF_3)) |
| *ompC* | GTAAAGAAGGGTAAA([2](#_ENREF_2)) |
| *ompF1* | GTAACCAAAAGTAAA([2](#_ENREF_2)) |
| *ompF2* | GTAATATTCCGTAAC([2](#_ENREF_2)) |
| *ppiA* | GTAAAATTAGGTAAA([2](#_ENREF_2)) |
| *psd* | GTAAAAACGCGTAAA([2](#_ENREF_2)) |
| *rseD* | GTAAAGTTCCGTAAC([2](#_ENREF_2)) |
| *skp* | GTAAAACCTGGTAAG([4](#_ENREF_4)) |
| *slt* | GTAACGTGGCGTAAA([2](#_ENREF_2)) |
| *spy* | GTAAAACAATGAAAG([2](#_ENREF_2)) |
| *srkA (yihE)* | GTAAAAGCTTGTAAG([2](#_ENREF_2)) |
| *tomB (ybaJ)* | GTAATTTTTCGTAAT([3](#_ENREF_3)) |
| *tsr* | GTAAAGTAAGGTAAA([2](#_ENREF_2)) |
| *ung* | GCAAAAAAATGTAAA([2](#_ENREF_2)) |
| *yccA* | GTAAAGATGGGTAAA([2](#_ENREF_2)) |
| *yebE* | GTATATCGAGACAAA([3](#_ENREF_3)) |
| *yidQ* | GTAAAGCGGGGATAA([4](#_ENREF_4)) |
| *yqaE* | GTAAATGAGAGTAAA([6](#_ENREF_6)) |

**Reference**

1. Hirakawa H, Inazumi Y, Masaki T, Hirata T, Yamaguchi A. 2005. Indole induces the expression of multidrug exporter genes in *Escherichia coli*. Mol Microbiol 55:1113-26.

2. De Wulf P, McGuire AM, Liu X, Lin EC. 2002. Genome-wide profiling of promoter recognition by the two-component response regulator CpxR-P in *Escherichia coli*. *J Biol Chem* 277:26652-61.

3. Yamamoto K, Ishihama A. 2006. Characterization of copper-inducible promoters regulated by CpxA/CpxR in *Escherichia coli*. Biosci Biotechnol Biochem 70:1688-95.

4. Dartigalongue C, Missiakas D, Raina S. 2001. Characterization of the *Escherichia coli* sigma E regulon. J Biol Chem 276:20866-75.

5. Xu Y, Zhao Z, Tong W, Ding Y, Liu B, Shi Y, Wang J, Sun S, Liu M, Wang Y, Qi Q, Xian M, Zhao G. 2020. An acid-tolerance response system protecting exponentially growing *Escherichia coli*. *Nat Commun* 11:1496.

6. Bernal-Cabas M, Ayala JA, Raivio TL. 2015. The Cpx envelope stress response modifies peptidoglycan cross-linking via the L,D-transpeptidase LdtD and the novel protein YgaU. J Bacteriol 197:603-14.

7. Weatherspoon-Griffin N, Yang D, Kong W, Hua Z, Shi Y. 2014. The CpxR/CpxA two-component regulatory system up-regulates the multidrug resistance cascade to facilitate *Escherichia coli* resistance to a model antimicrobial peptide. *J Biol Chem* 289:32571-82.
