# Supplementary material for: Systematic Identification of CpxRA-Regulated Genes and Their Roles in Escherichia coli Stress Response
Source: mSystems. 2022 Sep 7;7(5):e00419-22. doi: 10.1128/msystems.00419-22 (PMC9600279; doi:10.1128/msystems.00419-22)
Supplement: TABLE S4 [file msystems.00419-22-s0006.docx]

**Table S4　 Candidate CpxR-regulated genes obtained by position-specific scoring matrix**

| Gene |  | | Putative CpxR box | | | | | |  |
| --- | --- | --- | --- | --- | --- | --- | --- | --- | --- |
|  | Gene position | sequence | | sequence position | Distance*^a^* | spacer*^b^* | score*^c^* | |  |
| **multiple putative CpxR box exist within gene clusters** | | | | | | | | | |
| *carA-carB* | | | | | | | | | |
| *carA* | 29651..30799 | TTTGCattgaTTTAC | | 29579 | -58 | 5 | 15.89 | |  |
| *carB* | 30817..34038 | GTAATcaggaGTAAA | | 30796 | -7 | 5 | 11.53 | |  |
| *dusB-fis* | | | | | | | | | |
| *dusB* | 3403639..3404604 | TTTGCcgattaTTTAC | | 3403374 | -250 | 6 | 14.98 | |  |
| *fis* | 3404630..3404926 | GAAAAttttgcGTAAA | | 3404590 | -25 | 6 | 11.54 | |  |
| *yhdJ-yhdU* | | | | | | | | | |
| *yhdJ* | 3405012..3405896 | GTAAAaaattGAAAA | | 3404895 | -103 | 5 | 15.92 | |  |
| *yhdU* | 3405980..3406159 | GTAAAggatgtGTAAG | | 3405893 | -72 | 6 | 15.95 | |  |
| *frmA-frmB* | | | | | |  |  |  |  |
| *frmA* | 373918..375027 | GTAAAccgctGGAAA | | 374979 | 34 | 5 | 8.55 | |  |
| *frmB* | 372991..373824 | TTTCCcgcaggTTTAC | | 373902 | -78 | 6 | 8.49 | |  |
| *ygjR-alx* | | | | | |  |  |  |  |
| *ygjR* | 3230670..3231656 | GTAAAtacaaGTTAA | | 3230740 | 71 | 5 | 12.80 | |  |
| *alx* | 3231939..3232904 | GTAAAacagtGTAAA | | 3231665 | -260 | 5 | 19.10 | |  |
| **multiple putative CpxR box of exist at promoter regions of gene** | | | | | |  |  |  |  |
| *focA* | 949065..949922 | TTTACatataTTTGC | | 950238 | -316 | 5 | 14.93 | |  |
|  |  | TTTACtccgtaTTTGC | | 950039 | -117 | 6 | 12.84 | |  |
| *gudP* | 2914107..2915459 | GCAAAtgccaGTAAA | | 2915824 | -365 | 5 | 11.12 | |  |
|  |  | TTTACtggcgTTTGC | | 2915788 | -329 | 5 | 9.68 | |  |
| *fadI* | 4019887..4020588 | GTAAAaaaatgGTTAA | | 2453999 | -51 | 6 | 14.93 | |  |
|  |  | TTTACcgctgGTTAC | | 2453923 | 11 | 5 | 11.93 | |  |
| *shoB-ohsC* | | | | | | | | |  |
| *shoB* | 2693476..2693556 | TGTACttatctTTTAC | | 2693844 | -288 | 6 | 12.25 | |  |
|  |  | TTTGCtgtgcTTTAC | | 2693709 | -153 | 5 | 14.61 | |  |
| *ohsC* | 2693879..2693955 | GTAAAgcacaGCAAA | | 2693709 | -156 | 5 | 14.61 | |  |
|  |  | GTAAAagataaGTACA | | 2693844 | -20 | 6 | 12.25 | |  |
| *yibN-gpmM* | | | | | | | | |  |
| *yibN* | 3777944..3778375 | TTTGCgtaatTTTAC | | 3778576 | -201 | 5 | 16.03 | |  |
|  |  | TTTACgcttttTTTTC | | 3778490 | -115 | 6 | 14.00 | |  |
| *gpmM* | 3778620..3780164 | GAAAAaaaagcGTAAA | | 3778490 | -115 | 6 | 14.00 | |  |
|  |  | GTAAAattacGCAAA | | 3778576 | -30 | 5 | 16.03 | |  |
| *mtn-dgt* | | | | | | | | |  |
| *mtn* | 174942..175640 | GTAAGcgcatgGTAAA | | 175677 | -37 | 6 | 10.25 | |  |
|  |  | GTAAAtctatGAAAA | | 175634 | on | 5 | 15.69 | |  |
| *dgt* | 175724..177241 | TTTTCatagaTTTAC | | 175634 | -76 | 5 | 15.69 | |  |
|  |  | TTTACcatgcgCTTAC | | 175677 | -32 | 6 | 10.25 | |  |
| *prmB-smrB* | | | | | | | | |  |
| *prmB* | 2440987..2441919 | TTTTCctacagTTTAC | | 2442049 | -130 | 6 | 12.48 | |  |
|  |  | GTAAAtgagctGCAAA | | 2441877 | 27 | 6 | 12.91 | |  |
| *smrB* | 2442085..2442636 | TTTGCagctcaTTTAC | | 2441877 | -193 | 6 | 12.91 | |  |
|  |  | GTAAActgtagGAAAA | | 2442049 | -21 | 6 | 12.48 | |  |
| *lpxL-yceA* | | | | | | | | |  |
| *lpxL* | 1111118..1112038 | GGAAAtacttGTAAA | | 1112348 | -310 | 5 | 11.26 | |  |
|  |  | TTTACgctacaTTTGC | | 1112168 | -130 | 6 | 14.78 | |  |
| *yceA* | 1112263..1113315 | GCAAAtgtagcGTAAA | | 1112168 | -80 | 6 | 14.78 | |  |
|  |  | TTTACaagtaTTTCC | | 1112348 | 86 | 5 | 11.26 | |  |
| **Putative CpxR box exist at intergenic regions** | | | | | | | | |  |
| *chaB-chaA* | | | | | | | | |  |
| *chaB* | 1267575..1267805 | TTTACgaattTTTAC | | 1267393 | -168 | 5 | 17.88 | |  |
| *chaA* | 1266205..1267305 | GTAAAaattcGTAAA | | 1267393 | -88 | 5 | 17.88 | |  |
| *xthA-astC* | | | | | | | | |  |
| *xthA* | 1826685..1827491 | TTTACtggaaATTAC | | 1826511 | -160 | 5 | 11.36 | |  |
| *astC* | 1825019..1826239 | GTAATttccaGTAAA | | 1826511 | -272 | 5 | 11.36 | |  |
| *dasC-rhlB* | | | | | | | | |  |
| *dasC* | 3954037..3956058 | CTTACtattgcTTTAC | | 3959009 | -97 | 6 | 15.67 | |  |
| *rhlB* | 3957725..3958990 | GTAAAgcaataGTAAG | | 3959009 | -19 | 6 | 15.67 | |  |
| *ascF-ascG* | | | | | | | | |  |
| *ascF* | 2832883..2834340 | TTTTCatcccTTTAC | | 2832626 | -243 | 5 | 13.75 | |  |
| *ascG* | 2831613..2832623 | GTAAAgggatGAAAA | | 2832626 | -3 | 5 | 13.75 | |  |
| *ampH-sbmA* | | | | | | | | |  |
| *ampH* | 390586..391743 | TTTACgcgccGTTAC | | 391843 | -100 | 5 | 14.33 | |  |
| *sbmA* | 392095..393315 | GTAACggcgcGTAAA | | 391843 | -238 | 5 | 14.33 | |  |
| *copA-glsA* | | | | | | | | |  |
| *copA* | 504331..506835 | TTTACggactTTTAC | | 506926 | -91 | 5 | 16.68 | |  |
| *glsA* | 507097..508029 | GTAAAagtccGTAAA | | 506926 | -157 | 5 | 16.68 | |  |
| *araF-ftnB* | | | | | | | | |  |
| *araF* | 1978620..1979609 | TTTATatattTTTAC | | 1980212 | -603 | 5 | 15.16 | |  |
| *ftnB* | 1980406..1980909 | GTAAAaatatATAAA | | 1980212 | -180 | 5 | 15.16 | |  |
| *btsT-tsr* | | | | | | | | |  |
| *btsT* | 4578946..4581096 | TTTACcttacTTTAC | | 4581213 | -117 | 5 | 17.37 | |  |
| *tsr* | 4581474..4583129 | GTAAAgtaagGTAAA | | 4581213 | -247 | 5 | 17.37 | |  |
| *acnB-yacH* | | | | | | | | |  |
| *acnB* | 128102..130699 | TTAACgaagttTTTAC | | 127885 | -202 | 6 | 14.10 | |  |
| *yacH* | 125834..125848 | GTAAAaacttcGTTAA | | 127885 | -138 | 6 | 14.10 | |  |
| *qmcA-fetA* | | | | | | | | |  |
| *qmcA* | 510312..511229 | TTTTCtgattTTTAC | | 511349 | -120 | 5 | 14.90 | |  |
| *fetA* | 511375..512052 | GTAAAaatcaGAAAA | | 511349 | -12 | 5 | 14.90 | |  |
| *dsbG-ahpC* | | | | | | | | |  |
| *dsbG* | 633283..634029 | TTTACtcgactTTTGC | | 634052 | -23 | 6 | 13.27 | |  |
| *ahpC* | 634401..634964 | GCAAAagtcgaGTAAA | | 634052 | -334 | 6 | 13.27 | |  |
| *feaR-feaB* | | | | | | | | |  |
| *feaR* | 1440635..1441540 | TTTCCttattaTTTAC | | 1441756 | -216 | 6 | 11.58 | |  |
| *feaB* | 1441776..1443275 | GTAAAtaataaGGAAA | | 1441756 | -5 | 6 | 11.58 | |  |
| *ydeE-eamA* | | | | | | | |  |  |
| *ydeE* | 1615589..1616776 | TTTACttttctTTAAC | | 1615443 | -131 | 6 | 10.99 | |  |
| *eamA* | 1614495..1615394 | GTTAAagaaaaGTAAA | | 1615443 | -49 | 6 | 10.99 | |  |
| *exbB-metC* | | | | | | | | |  |
| *exbB* | 3144609..3145343 | GGAAAagaaaGCAAA | | 3145445 | -102 | 5 | 9.98 | |  |
| *metC* | 3145595..3146782 | TTTGCtttctTTTCC | | 3145445 | -136 | 5 | 9.98 | |  |
| *ygjH-ebgR* | | | | | | | | |  |
| *ygjH* | 3214274..3214606 | TTTAGtaaaatTTTAC | | 3214623 | -17 | 6 | 11.50 | |  |
| *ebgR* | 3214825..3215808 | GTAAAattttaCTAAA | | 3214623 | -187 | 6 | 11.50 | |  |
| *nrfA-acs* | | | | | | | | |  |
| *nrfA* | 4277692..4279128 | TTTACatgcaCTTAC | | 4277534 | -144 | 5 | 12.41 | |  |
| *acs* | 4275341..4277299 | GTAAGtgcatGTAAA | | 4277534 | -235 | 5 | 12.41 | |  |
| *fdnG-yddG* | | | | | | | | |  |
| *fdnG* | 1541658..1544705 | GTAATacccctGAAAA | | 1541630 | -13 | 6 | 11.39 | |  |
| *yddG* | 1540545..1541426 | TTTTCaggggtATTAC | | 1541630 | -204 | 6 | 11.39 | |  |
| *gmhA-fadE* | | | | | | | | |  |
| *gmhA* | 240030..240608 | TTTACaatataATTAC | | 239873 | -142 | 6 | 14.11 | |  |
| *fadE* | 237346..239790 | GTAATtatattGTAAA | | 239873 | -83 | 6 | 14.11 | |  |
| *rcsD-ompC* | | | | | | | | |  |
| *rcsD* | 2306967..2309639 | TTTACccttcTTTAC | | 2306653 | -300 | 5 | 18.34 | |  |
| *ompC* | 2305125..2306228 | GTAAAgaaggGTAAA | | 2306653 | -425 | 5 | 18.34 | |  |
| *ettA-slt* | | | | | | | | |  |
| *ettA* | 4618672..4620339 | TTTACgccacGTTAC | | 4620458 | -119 | 5 | 14.75 | |  |
| *slt* | 4620550..4622487 | GTAACgtggcGTAAA | | 4620458 | -78 | 5 | 14.75 | |  |
| *hyaA-yccA* | | | | | | | | |  |
| *hyaA* | 1027595..1028713 | TTTACccatcTTTAC | | 1026945 | -636 | 5 | 17.94 | |  |
| *yccA* | 1026215..1026874 | GTAAAgatggGTAAA | | 1026945 | -71 | 5 | 17.94 | |  |
| *yncD-yncE* | | | | | | | | |  |
| *yncD* | 1515220..1517322 | TTTACaatttcATTAC | | 1517361 | -39 | 6 | 14.90 | |  |
| *yncE* | 1517564..1518625 | GTAATgaaattGTAAA | | 1517361 | -188 | 6 | 14.90 | |  |
| *yfaH-inaA* | | | | | | | | |  |
| *yfaH* | 2343130..2343372 | TTTACccttcaTTTGC | | 2343070 | -45 | 6 | 14.47 | |  |
| *inaA* | 2342301..2342951 | GCAAAtgaaggGTAAA | | 2343070 | -119 | 6 | 14.47 | |  |
| *cybB-gapC* | | | | | | | | |  |
| *cybB* | 1485159..1485689 | TTTACtcatggTTTTC | | 1484962 | -182 | 6 | 10.02 | |  |
| *gapC* | 1483970..1484970 | GAAAAccatgaGTAAA | | 1484962 | on | 6 | 10.02 | |  |
| *amiC-argA* | | | | | | | | |  |
| *amiC* | 2941116..2942369 | TTTACgttccTTTAC | | 2942604 | -235 | 5 | 17.45 | |  |
| *argA* | 2942601..2943932 | GTAAAggaacGTAAA | | 2942604 | 4 | 5 | 17.45 | |  |
| *argE-argC* | | | | | | | | |  |
| *argE* | 4143624..4144775 | TTTATcgagaTTTAC | | 4144740 | 21 | 5 | 13.15 | |  |
| *argC* | 4144929..4145933 | GTAAAtctcgATAAA | | 4144740 | -175 | 5 | 13.15 | |  |
| *acpH-queA* | | | | | | | | |  |
| *acpH* | 419793..420374 | TTTCCggcaaTTTAC | | 420311 | 49 | 5 | 9.65 | |  |
| *queA* | 420467..421537 | GTAAAttgccGGAAA | | 420311 | -142 | 5 | 9.65 | |  |
| *plsB-dgkA* | | | | | | | | |  |
| *plsB* | 4243971..4246394 | TTTACtacaaATTAC | | 4246361 | 19 | 5 | 10.95 | |  |
| *dgkA* | 4246565..4246933 | GTAATttgtaGTAAA | | 4246361 | -190 | 5 | 10.95 | |  |
| **One putative CpxR box exist at promoter regions of gene** | | | | | | | | |  |
| *edd* | 1927050..1928861 | TTTATggttatTTTAC | | 1929065 | -204 | 6 | 14.99 | |  |
| *atpI* | 3915420..3915800 | TTTACgcgttaTTTAC | | 3916015 | -215 | 6 | 18.13 | |  |
| *cspA* | 3713409..3713621 | TTTATtgctgTTTAC | | 3713141 | -254 | 5 | 10.22 | |  |
| *adiA* | 4328071..4330338 | TTTTCacgcgcTTTAC | | 4330403 | -65 | 6 | 14.82 | |  |
| *cmoA* | 1946959..1947702 | TTTCCcggattTTTAC | | 1946940 | -4 | 6 | 11.02 | |  |
| *casA* | 2875989..2877497 | TTTTCtttgttTTTAC | | 2877564 | -67 | 6 | 15.63 | |  |
| *galP* | 3081643..3083037 | TTCACatcttTTTAC | | 3081574 | -55 | 5 | 11.49 | |  |
| *fimB* | 4530774..4531376 | TTTATgttgatTTTAC | | 4530452 | -307 | 6 | 14.70 | |  |
| *cbl* | 2053445..2054395 | TTTACcgtgaaTTTCC | | 2054386 | on | 6 | 11.04 | |  |
| *appC* | 1033196..1034740 | GTAAAaagacgGTAAG | | 1033059 | -122 | 6 | 15.57 | |  |
| *acpP* | 1147071..1147307 | GTAAAatcgtgGTAAG | | 1146966 | -90 | 6 | 13.46 | |  |
| *bluR* | 1208784..1209515 | GTAAAtcatcGTGAA | | 1209676 | -161 | 5 | 11.33 | |  |
| *cyoA* | 446119..447066 | GTAACcttcccGTAAA | | 447118 | -52 | 6 | 12.29 | |  |
| *gstA* | 1708634..1709239 | GTAAAattcagGGAAA | | 1708525 | -94 | 6 | 11.10 | |  |
| *frc* | 2485483..2486733 | GTATAttgagGTAAA | | 2487219 | -486 | 5 | 11.68 | |  |
| *xylE* | 4230707..4232182 | GTAAAcgcattGTAAA | | 4232264 | -82 | 6 | 13.61 | |  |
| *proP* | 4320319..4321821 | GTAAAtttggcGTAAA | | 4320041 | -263 | 6 | 16.75 | |  |
| *prlF* | 3270361..3270696 | GTAAAaggacaGTGAA | | 3270343 | -3 | 6 | 9.04 | |  |
| *hcp* | 907618..909270 | GTAAAacggcGGAAA | | 909180 | 76 | 5 | 12.35 | |  |
| *ecpR* | 306457..307047 | TTTACttccggTTTAC | | 307093 | -46 | 6 | 13.68 | |  |
| *epmB* | 4364446..4365474 | GTAAAaccggGTAAA | | 4365603 | -129 | 5 | 17.69 | |  |
| *yhdW* | 3412401..3413425 | GTAAAacacgcGTAAA | | 3412176 | -210 | 6 | 19.01 | |  |
| *ylaB* | 472523..474073 | GTAAAaatatcGTAAA | | 474265 | -192 | 6 | 18.49 | |  |
| *ybaL* | 497018..498694 | GTAAAatcagcGTAAA | | 499222 | -528 | 6 | 18.24 | |  |
| *atpB* | 3914596..3915411 | GTAAAtaacgcGTAAA | | 3916015 | -604 | 6 | 18.13 | |  |
| *sdaC* | 2921588..2922877 | TTTACttttgaTTTAC | | 2921349 | -224 | 6 | 17.81 | |  |

*^a^* The distance from putative CpxR box to the start codons of genes.

*^b^* The number of bases between two pentamers.

*^c^* the position-specific scoring matrix-recognition score.
