# Supplementary figures and images for: Systematic Identification of CpxRA-Regulated Genes and Their Roles in Escherichia coli Stress Response
Source: mSystems. 2022 Sep 7;7(5):e00419-22. doi: 10.1128/msystems.00419-22 (PMC9600279; doi:10.1128/msystems.00419-22)

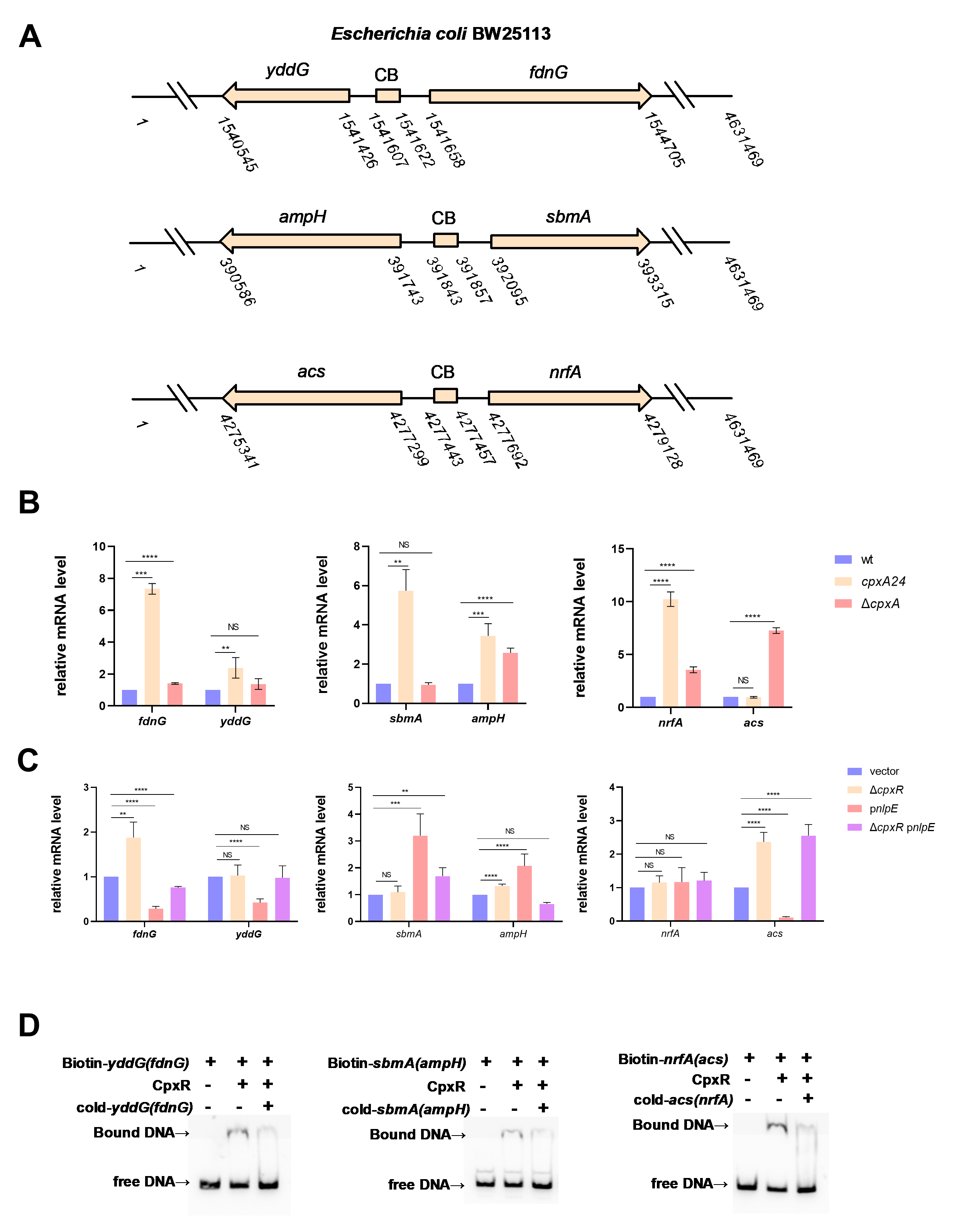

Supplement: FIG S1 [file msystems.00419-22-s0002.tif]

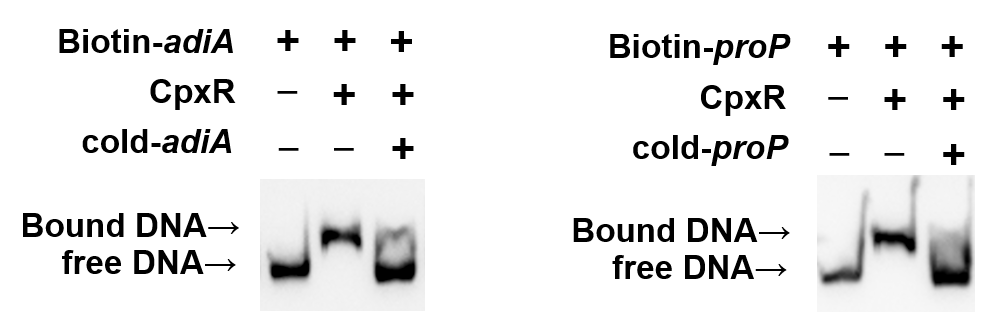

Supplement: FIG S2 [file msystems.00419-22-s0003.tif]

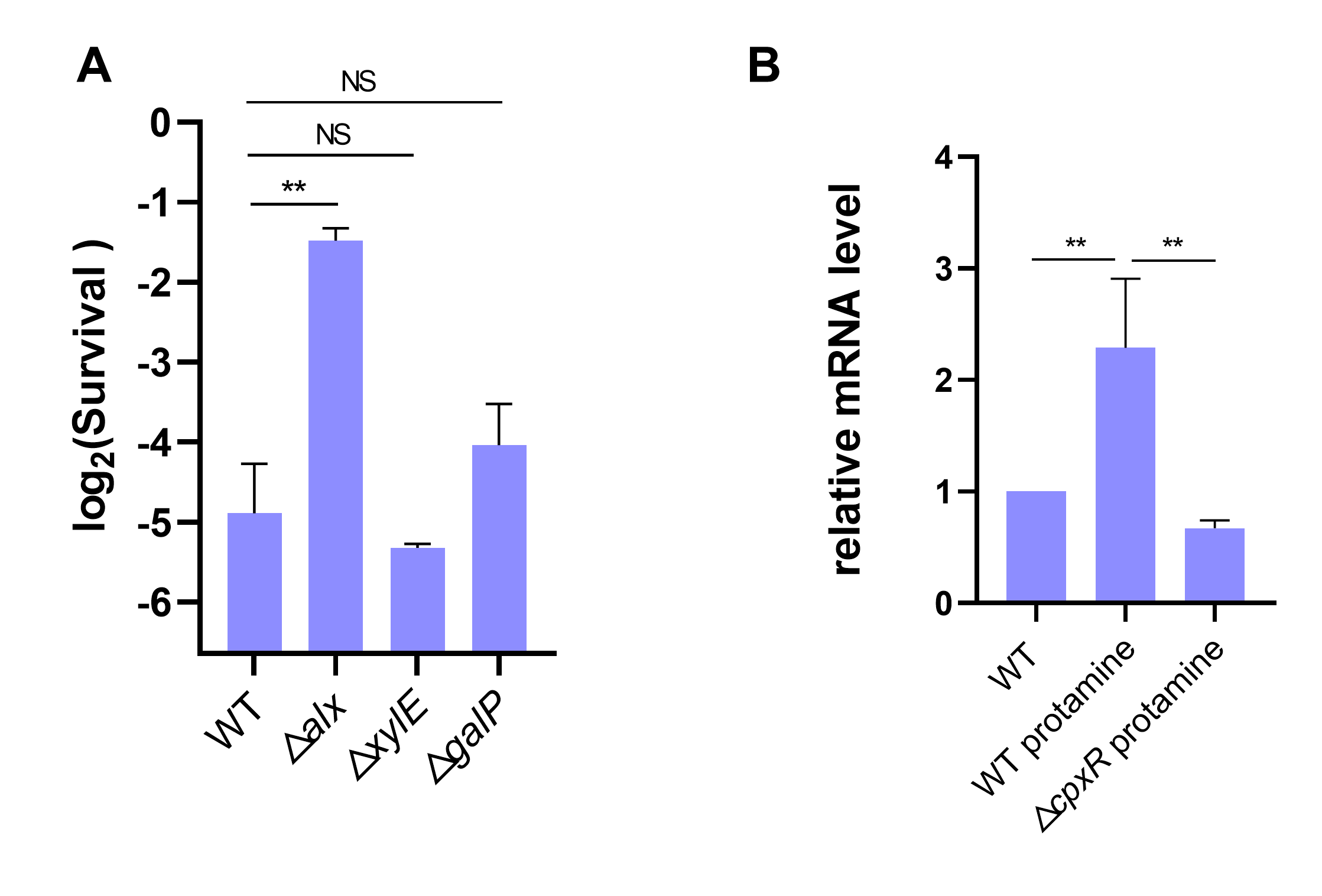

Supplement: FIG S3 [file msystems.00419-22-s0004.tif]
