# Supplementary material for: Systematic Identification of CpxRA-Regulated Genes and Their Roles in Escherichia coli Stress Response
Source: mSystems. 2022 Sep 7;7(5):e00419-22. doi: 10.1128/msystems.00419-22 (PMC9600279; doi:10.1128/msystems.00419-22)
Supplement: TABLE S5 [file msystems.00419-22-s0007.docx]

Table S5　Strain and plasmid used in this study

| Strains and plasmids | Description | Source |
| --- | --- | --- |
| ***E. coli* strains** |  |  |
| DH5α | F^–^ *supE*44 Δ*lacU*169 (*ϕ*80 *lacZ* Δ*M15*) *hsdR*17 *recA*1 *endA1 gyrA*96 *thi*-1 *relA*1 | lab collection |
| BW25113 | *rrnB3 ΔlacZ4787 hsdR514* Δ(*araBAD*)*567* Δ(*rhaBAD)568 rph-1* | lab collection |
| Q2576 | *E. coli* χ7213/pRE112*-cpxA24* | lab collection |
| Q1419 | *E. coli* BW25113 | lab collection |
| Q3172 | *E. coli* BW25113 *cpxA24* | This study |
| Q1821 | *E. coli* BW25113*△cpxA::Kan* | lab collection |
| Q1750 | *E. coli* BW25113*△cpxR::Kan* | lab collection |
| Q1614 | *E. coli* BW25113*/*pTrcHis2B | lab collection |
| Q1696 | *E. coli* BW25113*/*pTrcHis2B*-nlpE* | lab collection |
| Q1938 | *E. coli* BW25113*△cpxR::Kan/*pTrcHis2B*-nlpE* | lab collection |
| Q1442 | *E. coli* BW25141/pKD3 | lab collection |
| Q1461 | *E. coli* BW25113*/*pKD46 | lab collection |
| Q3611 | *E. coli* BW25113*△cpxR△cpxA::Cm* | This study |
| Q3613 | *E. coli* BW25113*△cpxA::Kan;△ackA-pta::Cm* | This study |
| Q3615 | *E. coli* BW25113*△cpxR::Kan; ompR::Cm/* pTrcHis2B*-nlpE* | This study |
| Q1751 | *E. coli* BW25113*/*pTrcHis2B*-cpxR* | lab collection |
| Q2605 | *E. coli* BW25113*/*pTrcHis2B*-cpxA* | lab collection |
| Q4308 | *E. coli* BW25113*△cpxR::Kan/*pTrcHis2B | This study |
| Q4317 | *E. coli* BW25113*△ackA-pta::Cm* | This study |
| **Plasmids** |  |  |
| pKD46 | rep_pSC101_^ts^ Ap^R^ P_araBAD_γβexo | lab collection |
| pTrcHis2B | rep_pBR322_ Ap^R^ *lacI*^q^ P*_trc_* | Invitrogen |
| pTrcHis2B-*nlpE* | rep_pBR322_ Ap^R^ *lacI*^q^ P*_trc_nlpE* | lab collection |
| pRE112 | *oriT oriV sacB* Cm^R^ | Invitrogen |
