# Supplementary material for: Systematic Identification of CpxRA-Regulated Genes and Their Roles in Escherichia coli Stress Response
Source: mSystems. 2022 Sep 7;7(5):e00419-22. doi: 10.1128/msystems.00419-22 (PMC9600279; doi:10.1128/msystems.00419-22)
Supplement: TABLE S6 [file msystems.00419-22-s0008.docx]

Table S6　Primers used in this study

| Primers | Nucleotide Sequence (5’-3’) |
| --- | --- |
| **Reverse transcription PCR** | |
| *chaA* coding region in *E. coli* | |
| 1929 | TATTGTTGGGCGGTCGTAA |
| 1930 | CAGGCAGAGCCATTGGAAA |
| *chaB* coding region in *E. coli* | |
| 1931 | AGCACGTTCTACCGTCTCATG |
| 1932 | CGTCATCACGCCGATCTTCT |
| *atpI* coding region in *E. coli* | |
| 1935 | CTGTTCAGCCTCAAAGACCCC |
| 1936 | TGGTGACGCCAGGCAAATA |
| *xylE* coding region in *E. coli* | |
| 1937 | AAATTATCGGCGCACTCGG |
| 1938 | TGGCAAAGGCGGCAACAT |
| *proP* coding region in *E. coli* | |
| 1939 | TTATCGCTCTGCCGTTAGGG |
| 1940 | CGAGACTTTCGGGCCATCC |
| *rcsD* coding region in *E. coli* | |
| 1941 | CCCCGGAAAGCAAACAAC |
| 1942 | GGCAACACTGAAGGCACAAC |
| *ompC* coding region in *E. coli* | |
| 1943 | GGCTTCAAAGGTGAAACTCAG |
| 1944 | GTAGTCGAAAGAACCCACATCC |
| *degP* coding region in *E. coli* | |
| 1947 | GCACTGGCTCTGAGTTTAGGTT |
| 1948 | ATCTGCTGGGCTGTCGTTG |
| *ppiA* coding region in *E. coli* | |
| 1949 | CTTTCTCCCGCAGCAATGG |
| 1950 | AAACCGCTGTTCACATAATCGA |
| *yncD* coding region in *E. coli* | |
| 1967 | CACCACAACTTAACCCGTCACA |
| 1968 | TGTGTCCAGCGGCTGTCTATT |
| *yncE* coding region in *E. coli* | |
| 1969 | ATTCTGGCGAAGGTTGCG |
| 1970 | TGTTTGGATGAGTCGGCGTAT |
| *inaA* coding region in *E. coli* | |
| 1971 | CGCATCATCTGTTTCATTCCG |
| 1972 | GCTCGCCATTCACCCTCAA |
| *prlF* coding region in *E. coli* | |
| 1975 | CTGGGAGATGAACAGGAGGAT |
| 1976 | CATGCCAGCGACAAGTTTC |
| *srkA* coding region in *E. coli* | |
| 1981 | GGAACACGCTCCAAATGACC |
| 1982 | TTCGAAGTATGCCTCCAACG |
| *carA* coding region in *E. coli* | |
| 1983 | CTGCGGATGCTGGTGGAT |
| 1984 | TTCTGGATGGCGGTAATGG |
| *carB* coding region in *E. coli* | |
| 1985 | GCACTGGCTCTGAGTTTAGGTT |
| 1986 | ATCTGCTGGGCTGTCGTTG |
| *frmB* coding region in *E. coli* | |
| 1987 | CGGTGCTGTACTGGCTTTCA |
| 1988 | GTCCGGCGCAACAACAAT |
| *frmA* coding region in *E. coli* | |
| 1989 | AAACCTGGCGACCATGTGA |
| 1990 | CGGCTGCCCGTTGTAAGA |
| *ygjR* coding region in *E. coli* | |
| 2579 | AGGGCGTGGTGGTGATGGA |
| 2580 | TGCTGCGGCTGGGTGAGAT |
| *alx* coding region in *E. coli* | |
| 1993 | CTGGTGCAAACCGAAGGTC |
| 1994 | CAACATCAGCCAGACAAAGACG |
| *dusB* coding region in *E. coli* | |
| 1995 | GGAACACGCTCCAAATGACC |
| 1996 | TTTCGAAGTATGCCTCCAACG |
| *fis* coding region in *E. coli* | |
| 1997 | CCCTGCGTGACTCGGTTAAA |
| 1998 | CGCAGCACGGGTCTGGTTA |
| *yhdJ* coding region in *E. coli* | |
| 1999 | TGGTATTGAGATCAACAGCGAGTA |
| 2000 | GCCAGTTCTTCCGCAGAGTA |
| *yhdU* coding region in *E. coli* | |
| 2001 | TTCGCAAGTATTGGTGGCTC |
| 2002 | TTGCGGCATTTGTCTGTTTC |
| *lpxL* coding region in *E. coli* | |
| 2009 | GGCAGTTTGGTATGCAGGAA |
| 2010 | CCCAGGTTTGTAGCCAGTCA |
| *yceA* coding region in *E. coli* | |
| 2011 | ATGGCTGCCACCTGCTGT |
| 2012 | CGGCGTCGCTGTTCTTCT |
| *prmB* coding region in *E. coli* | |
| 2013 | ACGAATACCGCCACGAGC |
| 2014 | CTTCACAAATCAACACGCCATC |
| *smrB* coding region in *E. coli* | |
| 2015 | AAACAACACTTAGCGAGGAGGA |
| 2016 | TGCGGTCGGTGGACAATC |
| *gudP* coding region in *E. coli* | |
| 2019 | GCACAAATGCTCGTTACTGGAT |
| 2020 | TTTCCGAACCGGCGATAGA |
| *yibN* coding region in *E. coli* | |
| 2023 | TGTTGCCGAGCGAAATCA |
| 2024 | ATGCCAGAACCGTCTACCAC |
| *gpmM* coding region in *E. coli* | |
| 2025 | TCTGCAACCGGAAATGAGC |
| 2026 | ATGTCGCCGTTCGGATAGTT |
| *mtn* coding region in *E. coli* | |
| 2573 | TCTGAAATCGGGCATCGGT |
| 2574 | GGTGTTAATAATCACATCTGGCTTG |
| *dgt* coding region in *E. coli* | |
| 2571 | GGCAATCCGCCGTTTGGT |
| 2572 | AGTCAGAGGCTGGCTTTCG |
| *focA* coding region in *E. coli* | |
| 2031 | CCGCAAATGGTCAATGGG |
| 2032 | TGCCAGGATACCAAGACAGACG |
| *acnB* coding region in *E. coli* | |
| 2061 | GTGACTCCCATACCCGTTTCC |
| 2062 | GGCATATCAAGCGGCATTAC |
| *acpH* coding region in *E. coli* | |
| 2063 | CCGGCAATTTACTGGCTGAT |
| 2064 | AAACCACTCCCGTGCTTCG |
| *acpP* coding region in *E. coli* | |
| 2065 | GCTGGTAATGGCTCTGGAAG |
| 2066 | CCTGGTGGCCGTTGATGTA |
| *acs* coding region in *E. coli* | |
| 2067 | AAGCGAGCGATCAGCACC |
| 2068 | CGCCGCGTACACCAGATAA |
| *nrfA* coding region in *E. coli* | |
| 2569 | TGCGTGAAACCCTGCGTAC |
| 2570 | TGGCAATCGGCACAACCTA |
| *adiA* coding region in *E. coli* | |
| 2069 | GCTGGTAATGGCTCTGGAAG |
| 2070 | CCTGGTGGCCGTTGATGTA |
| *ahpC* coding region in *E. coli* | |
| 2071 | CCACAAAGCATGGCACAGC |
| 2072 | AACGAAGGTCGCACGGTCA |
| *amiC* coding region in *E. coli* | |
| 2073 | GCTGGTAATGGCTCTGGAAG |
| 2074 | CCTGGTGGCCGTTGATGTA |
| *ampH* coding region in *E. coli* | |
| 2075 | GCGAGCAACGCTGGAAAT |
| 2076 | AGCAGGTCGAACGCAAGG |
| *appC* coding region in *E. coli* | |
| 2077 | ATTTGATGGCTGAAACCTACCC |
| 2078 | GGCTCACGATTGCCTTGC |
| *araF* coding region in *E. coli* | |
| 2079 | GGAAGGATTTAGGGTTTGAGGT |
| 2080 | TGGCAGAGCCGAGTTTGG |
| *argA* coding region in *E. coli* | |
| 2081 | CTTCCCAGGGCGTCACTAA |
| 2082 | CGGAGTTGTAATCGCCTTTC |
| *argC* coding region in *E. coli* | |
| 2083 | GGAAGGATTTAGGGTTTGAGGT |
| 2084 | TGGCAGAGCCGAGTTTGG |
| *argE* coding region in *E. coli* | |
| 2085 | GGAAGGATTTAGGGTTTGAGGT |
| 2086 | TGGCAGAGCCGAGTTTGG |
| *ascF* coding region in *E. coli* | |
| 2087 | AGCATCGTCTGGGTGTTCG |
| 2088 | GTCGGTTGTACGCTCTGATACTTT |
| *ascG* coding region in *E. coli* | |
| 2089 | GACCAACACGCTTTACCACG |
| 2090 | AGCAACTGCCGCCCTTTC |
| *astC* coding region in *E. coli* | |
| 2091 | ATTTGATGGCTGAAACCTACCC |
| 2092 | GGCTCACGATTGCCTTGC |
| *bluR* coding region in *E. coli* | |
| 2093 | GCGGACACCGACTCTTTGAT |
| 2094 | CCTACAGGGACGCCATTACTT |
| *btsT* coding region in *E. coli* | |
| 2095 | GCCTAACCTGCATGAAATGGG |
| 2096 | CAGGATCTGCTCTGGCGAAAT |
| *casA* coding region in *E. coli* | |
| 2097 | CCTTAATCACGCAGAACATCCC |
| 2098 | CCACCACATAATGCTTCACCCT |
| *cbl* coding region in *E. coli* | |
| 2099 | GCCGTTAATCACTTACCGACAG |
| 2100 | ATCCCAAGCCCAAGAGCA |
| *cmoA* coding region in *E. coli* | |
| 2101 | TTGTTCAACATGCACCACGAC |
| 2102 | GGAATCGGTCAGCATCACG |
| *copA* coding region in *E. coli* | |
| 2103 | GACCAACACGCTTTACCACG |
| 2104 | AGCAACTGCCGCCCTTTC |
| *cpxP* coding region in *E. coli* | |
| 2105 | GCTCGTCAGGTTGAGATGGC |
| 2106 | CGTCACGCAACTGCTCCATT |
| *cspA* coding region in *E. coli* | |
| 2107 | GCTTCGGCTTCATCACTCCT |
| 2108 | GACACTTTCTGACCTTCGTCCA |
| *cybB* coding region in *E. coli* | |
| 2109 | ATGTTTCCTGTGGCATCTCAAT |
| 2110 | GTCCCGTCATCATCGGTTTT |
| *cyoA* coding region in *E. coli* | |
| 2111 | GGAACAGGGCATTGCTACCG |
| 2112 | GAGTTCATCACGGAGTTGGAGGT |
| *dasC* coding region in *E. coli* | |
| 2115 | GTGGTATCCCGACTCTGCTGC |
| 2116 | GGTTAGCGTCGAGGAACTCTTTC |
| *dgkA* coding region in *E. coli* | |
| 2117 | AGGAAGGCGTAGCGGTATTGTT |
| 2118 | CATCACCAGCATCACGGAGC |
| *plsB* coding region in *E. coli* | |
| 2577 | ACTTGCCTGACCCGTTAGAGC |
| 2578 | CTTCTTTCGGCGTGTAATAGGTG |
| *dsbG* coding region in *E. coli* | |
| 2121 | AGCGATTGAAAAACAGGGCA |
| 2122 | CTGGAGTCAGGTAGATGGTGACG |
| *eamA* coding region in *E. coli* | |
| 2123 | AGCAGCATTTAGTTGGGCGTG |
| 2124 | AACCATCGAGAATCAGCGAGG |
| *ebgR* coding region in *E. coli* | |
| 2125 | TATGGCCGACTGAAACAAGTGG |
| 2126 | AGTACGCCGATAGCAATGGAA |
| *ecpR* coding region in *E. coli* | |
| 2127 | AGTGATTACAGTAGGGACTATGAGGTT |
| 2128 | CAGATAGTCCTTTATAGAAGTAGGCGT |
| *edd* coding region in *E. coli* | |
| 2129 | TCGCCATCATCACCTCCTATAA |
| 2130 | GCGACAATTCCATTCCATCC |
| *efeU* coding region in *E. coli* | |
| 2131 | TGTGTGATGTGGATTGGCGT |
| 2132 | CAGTTCCTGTTCTTTTTGCGG |
| *ettA* coding region in *E. coli* | |
| 2133 | CGATTGCTGCTGTTGGTGCT |
| 2134 | GGAAGTAATATGGATGCCGTGCT |
| *exbB* coding region in *E. coli* | |
| 2135 | AGCGATTGAAAAACAGGGCA |
| 2136 | CTGGAGTCAGGTAGATGGTGACG |
| *fadE* coding region in *E. coli* | |
| 2137 | CCTGCCTTCCAACTCAACC |
| 2138 | CAATCCCTTCCATCTTACCAAT |
| *fadI* coding region in *E. coli* | |
| 2139 | CGGTTTACGTACGCCTTTTG |
| 2140 | AGTTCGCCTACCACCATCTTC |
| *fdnG* coding region in *E. coli* | |
| 2581 | CGTAACGACGGTGAAATTCTG |
| 2582 | GTTCGTGCGGCTGCTTGT |
| *feaB* coding region in *E. coli* | |
| 2143 | GCCGGAGAGGGGTATTATGTT |
| 2144 | CCGTGTCGTTTGCCAGTTGT |
| *feaR* coding region in *E. coli* | |
| 2145 | CAGGAAGTAAAAGGCAGCGAC |
| 2146 | CAGTAAAGCGAACAGGGGC |
| *fetA* coding region in *E. coli* | |
| 2147 | CAGCCTGACCCAGCCATTT |
| 2148 | TGCGTTGTTTTTCACCACCA |
| *fimB* coding region in *E. coli* | |
| 2149 | AGGCTTTTCAACAACGCACC |
| 2150 | GATAAAAATACCCACTCGCTCTCAG |
| *frc* coding region in *E. coli* | |
| 2151 | AATACCAAAACAGCGGAAGGC |
| 2152 | CCTTTGATCGAACCAAAAATCAG |
| *ftnB* coding region in *E. coli* | |
| 2153 | ATGGCAACCGCTGGAATG |
| 2154 | CTGGGCGCGAAGGAAAGT |
| *galP* coding region in *E. coli* | |
| 2155 | TAAACTCGGGCGCAAAAAGA |
| 2156 | CCCAGTAGAACGCGGGAAA |
| *gapC* coding region in *E. coli* | |
| 2157 | TCGGTCGACTGGTGTTGGG |
| 2158 | AGGCGAGAATTTATGGGGAAG |
| *glsA* coding region in *E. coli* | |
| 2161 | CAGTGCTCCACGCTCCTCAA |
| 2162 | CCCTTCCATCATCATTTCGG |
| *gstA* coding region in *E. coli* | |
| 2171 | TTCACACCTCTGTTTCGCCC |
| 2172 | CTCATCCTTCAGTGCCTCGTT |
| *hcp* coding region in *E. coli* | |
| 2173 | CTGCCTGGGCGGTAAAAG |
| 2174 | CACGAGCGTAGCCGACAATA |
| *htpX* coding region in *E. coli* | |
| 2179 | TGCTGTTCGGTTTTGGTGGT |
| 2180 | GTTCCCTTTCGTTACGCGGT |
| *hyaA* coding region in *E. coli* | |
| 2181 | GGAATGGCACCAAAGATTGC |
| 2182 | GTGAGCGGAGCGGATAAAAG |
| *yacH* coding region in *E. coli* | |
| 2190 | GCTTTGTACGCGGATTCGG |
| 2191 | CGTTGTGTTGCCAGCCATT |
| *sbmA* coding region in *E. coli* | |
| 2228 | GTTTGTTCTTGCTGTTTCCGTC |
| 2229 | CAACCAGTGTGGTCCATGAGTT |
| *queA* coding region in *E. coli* | |
| 2196 | CGAACTTCCACTTGCCAGAG |
| 2197 | ATTTCTCTTCTACCGCTGCTTT |
| *qmcA* coding region in *E. coli* | |
| 2198 | CAGTCTGGTGGTGCCGTTTA |
| 2199 | ACGTTGGCGTTATCTTTCGA |
| *yccA* coding region in *E. coli* | |
| 2200 | CCTCGGACCTATTCTGAACACC |
| 2201 | CCGAGGAACGACATATCTTTGC |
| *yddG* coding region in *E. coli* | |
| 2202 | GGCCCAGCCTGACAATTCT |
| 2203 | TCACCGCCTAACACCCAAC |
| *shoB* coding region in *E. coli* | |
| 2204 | ATGACTGATTGCCGATACCTGA |
| 2205 | CACAAGGATCAGCTGTAAAACAGC |
| *ohsC* coding region in *E. coli* | |
| 2575 | GTTGAGGGTGCATGCTGCACA |
| 2576 | AACCCCCGAACTGGTAAGGAAC |
| *metC* coding region in *E. coli* | |
| 2208 | AGGTGTGCGTTTGCGTCAA |
| 2209 | CAGGCAGAGCAGGGTGGTT |
| *ydeE* coding region in *E. coli* | |
| 2210 | TGACGCTGGTTGTGCTCTTT |
| 2211 | GATTTTCGTTTTGCTGGTGGA |
| *xthA* coding region in *E. coli* | |
| 2212 | AGGCTGATGAGCTGGGGGT |
| 2213 | CGTCAAAACCTTTTGAGCGGTA |
| *ygjH* coding region in *E. coli* | |
| 2214 | CGTGGCTTACGCTGATTTTG |
| 2215 | CTGTAGTACGGCACCAGGCTG |
| *rhlB* coding region in *E. coli* | |
| 2216 | AGGTGGTGGTACTGGACGAA |
| 2217 | GGCGGAGAACAGCATGTTG |
| *tsr* coding region in *E. coli* | |
| 2226 | GCAGGCAAAATCAACGAGTTC |
| 2227 | CGCCCACCAGAATCCACAT |
| *slt* coding region in *E. coli* | |
| 2220 | GCGTGGCTTGTTAGCCTTTAG |
| 2221 | TCTTCACTCTGCCCGGTGTT |
| *gmhA* coding region in *E. coli* | |
| 2230 | CTGCTGGGGATCTCCACCT |
| 2231 | CATTTTGCCGCCGTCTTTAC |
| *yfaH* coding region in *E. coli* | |
| 2232 | TTGTGTTCCGTTCCTCTCGA |
| 2233 | CAATCGCCACTAATTGTTCCAT |
| *psd* coding region in *E. coli* | |
| 3114 | CAAGGTCGACATGAAAGAGGC |
| 3115 | GCAGCGGACGGACAAAGAAT |
| *motA* coding region in *E. coli* | |
| 3116 | TCCTCGGCATTTTATTGGCTT |
| 3117 | CACTGCATCATTTTGCTGGTTTC |
| *empB* coding region in *E. coli* | |
| 3120 | GAATATAGACGCGGAGGAAAAAC |
| 3121 | GATAAATGAGCGGGGCACAC |
| *yhdW* coding region in *E. coli* | |
| 3122 | CAGGGGAGGTGGATTTGCT |
| 3123 | GCTTTATCGTGCGTCAGGAA |
| *ylaB* coding region in *E. coli* | |
| 3124 | TTTCTGAGCCATTGACGCTACTG |
| 3125 | CGGGATTTTTTCCGAGGTG |
| *ybaL* coding region in *E. coli* |  |
| 3126 | GGTACTGTTCGCACTACTGGAGA |
| 3127 | CCACTGGGATCTGCTTCTCTTCT |
| *atpB* coding region in *E. coli* |  |
| 3128 | CGCAGGATTACATAGGACACCA |
| 3129 | GCTACGGAATAAAACCAGGAACAA |
| *sdaC* coding region in *E. coli* |  |
| 3130 | AGAACAGAAATGCTCGAAGATCC |
| 3131 | GGCTCAGTACACAGCTGAAGACG |
| **Construction of *cpxA24* mutant strains** | |
| 1309 | GATAAGTGGGCACCGCCAG |
| 1310 | gtaattatcttcgccatc |
| **Construction of *E. coli* BW25113Δ*cpxR*Δ*cpxA*** | |
| 2462 | AGCGACGTCTGATGACGTAATTTCTGCCTCGGAGGTATTTAAACgtgtaggctggagctgcttc |
| 2463 | AAATGCCGGATGCGGCGTAAACGCCTTATCCTGCCTGCAAATGCGAAGTcatatgaatatcctccttag |
| 1607 | GAACATATGGCTCTGCGTAC |
| 864 | AGTTTCTACACATATATTCG |
| **Construction of *E. coli* BW25113Δ*cpxR*Δ*ompR*** | |
| 2464 | GAATACACGCTTACAAATTGTTGCGAACCTTTGGGAGTACAAACAgtgtaggctggagctgcttc |
| 2465 | AATAACGTACGGGCAAATGAACTTCGTGGCGAGAAGCGCAATCGCCcatatgaatatcctccttag |
| 2466 | GTCTGTTTGATAATGCGCAC |
| 864 | AGTTTCTACACATATATTCG |
| **Construction of *E. coli* BW25113ΔcpxAΔ*pta*Δ*ackA*** | |
| 2467 | GGCTCCCTGACGTTTTTTTAGCCACGTATCAATTATAGGTACTTCCgtgtaggctggagctgcttc |
| 2468 | CCGGTTCAGATATCCGCAGCGCAAAGCTGCGGATGATGACGAGAcatatgaatatcctccttag |
| 2469 | CATGCTTCACCTCAACTTCAC |
| 864 | AGTTTCTACACATATATTCG |
| **Construction of *E. coli* BW25113Δ*pta*Δ*ackA*** | |
| 2467 | GGCTCCCTGACGTTTTTTTAGCCACGTATCAATTATAGGTACTTCCgtgtaggctggagctgcttc |
| 2468 | CCGGTTCAGATATCCGCAGCGCAAAGCTGCGGATGATGACGAGAcatatgaatatcctccttag |
| 2469 | CATGCTTCACCTCAACTTCAC |
| 864 | AGTTTCTACACATATATTCG |
| **EMSA** | |
| CB1 of *carA-carB* | |
| 2532 | CCCATATCTCCAGAATGC |
| 2533 | GACTTAATCAAAACACCCTC |
| CB2 of *carA-carB* | |
| 2534 | CGTTGTTCGACCACTTTATCG |
| 2535 | GCACCCAGAATCAGGATAC |
| CB1 of *shoB-ohsC* | |
| 2536 | TTGACGACACCACTCGTTTC |
| 2537 | GACCGCCAATTCGTAAGTAC |
| CB2 of *shoB-ohsC* | |
| 2538 | GCTTTCTTCGTAAAGGCTCG |
| 2539 | CAGCATGCACCCTCAACAAC |
| CB1 of *dusB-fis* | |
| 2561 | GCAAGCTCACAAAAGGCACG |
| 2562 | GCGTGGTGCGATAATTACTC |
| CB2 of *dusB-fis* | |
| 2563 | TTGAGGATGCCAGCGAACAG |
| 2564 | ACGTCAGAATTTACGCGTTG |
| *chaA-chaB* | |
| 2531 | CAGACTCTTCTTAAGAGACG |
| 2266 | CGGGTTTTTACCGCCTCTTG |
| *yddG-fdnG* | |
| 2547 | CTGCGACTGACGTCCATTGC |
| 2548 | GAAGGCGGATTATTTTGTGG |
| *ampH-sbmA* | |
| 2549 | AAGCGGATTAACACGCGCAG |
| 2550 | GATTGCAAAGGGCGAATTAG |
| *acs-nrfA* | |
| 2551 | ATGCAGTTATGCATGCTGTTG |
| 2551 | GCTCGCCCCTATGTGTAAC |
| *xylE* |  |
| 2530 | GCTCTGAGTCACGGCAATAG |
| 2258 | CATTCAGACCTGCCTTAGACCAT |
| *proP* | |
| 3132 | TGGACCACGGCAGATAAACAAC |
| 3133 | GAGCGGGAATTGCAGGATG |
| *adiA* | |
| 3134 | ACCGCAAACTTAGCCAACAGC |
| 3135 | GATGGAGAAACTCGCTTTCAACA |
